# Glutaminase-containing microvesicles from HIV-1-infected macrophages and immune-activated microglia induce neurotoxicity

**DOI:** 10.1186/s13024-015-0058-z

**Published:** 2015-11-06

**Authors:** Beiqing Wu, Yunlong Huang, Alexander L. Braun, Zenghan Tong, Runze Zhao, Yuju Li, Fang Liu, Jialin C. Zheng

**Affiliations:** Laboratory of Neuroimmunology and Regenerative Therapy, Departments of Pharmacology and Experimental Neuroscience, University of Nebraska Medical Center, Omaha, NE 68198-5930 USA; Departments of Pathology and Microbiology, University of Nebraska Medical Center, Omaha, NE 68198-5930 USA; Center for Translational Neurodegeneration and Regenerative Therapy, Shanghai Tenth People’s Hospital, Tongji University School of Medicine, Shanghai, 200025 China

**Keywords:** HAND, HIV, Glutamate, Glutaminase, Microvesicles, Neurotoxicity

## Abstract

**Background:**

HIV-1-infected and/or immune-activated microglia and macrophages are pivotal in the pathogenesis of HIV-1-associated neurocognitive disorders (HAND). Glutaminase, a metabolic enzyme that facilitates glutamate generation, is upregulated and may play a pathogenic role in HAND. Our previous studies have demonstrated that glutaminase is released to the extracellular fluid during HIV-1 infection and neuroinflammation. However, key molecular mechanisms that regulate glutaminase release remain unknown. Recent advances in understanding intercellular trafficking have identified microvesicles (MVs) as a novel means of shedding cellular contents. We posit that during HIV-1 infection and immune activation, microvesicles may mediate glutaminase release, generating excessive and neurotoxic levels of glutamate.

**Results:**

MVs isolated through differential centrifugation from cell-free supernatants of monocyte-derived macrophages (MDM) and BV2 microglia cell lines were first confirmed in electron microscopy and immunoblotting. As expected, we found elevated number of MVs, glutaminase immunoreactivities, as well as glutaminase enzyme activity in the supernatants of HIV-1 infected MDM and lipopolysaccharide (LPS)-activated microglia when compared with controls. The elevated glutaminase was blocked by GW4869, a neutral sphingomyelinase inhibitor known to inhibit MVs release, suggesting a critical role of MVs in mediating glutaminase release. More importantly, MVs from HIV-1-infected MDM and LPS-activated microglia induced significant neuronal injury in rat cortical neuron cultures. The MV neurotoxicity was blocked by a glutaminase inhibitor or GW4869, suggesting that the neurotoxic potential of HIV-1-infected MDM and LPS-activated microglia is dependent on the glutaminase-containing MVs.

**Conclusions:**

These findings support MVs as a potential pathway/mechanism of excessive glutamate generation and neurotoxicity in HAND and therefore MVs may serve as a novel therapeutic target.

**Electronic supplementary material:**

The online version of this article (doi:10.1186/s13024-015-0058-z) contains supplementary material, which is available to authorized users.

## Background

HIV-1-associated neurocognitive disorders (HAND) are currently prevalent in spite of major advances in combination anti-retroviral therapy. Therefore, novel therapeutic targets are required to be developed to treat the disease [1–4]. The HIV-1-infected and immune-activated mononuclear phagocytes (MPs, including macrophages and microglia) are critical to HAND pathogenesis, producing a variety of inflammatory and neurotoxic factors, including excess levels of the excitatory neurotransmitter glutamate [5, 6]. Glutamate is a major mediator of excitatory synaptic transmission and has a vital role in mediating learning and memory [7–9]. Early studies reported that the concentrations of glutamate in the plasma and cerebrospinal fluid were significantly higher in HIV-1-infected patients than in uninfected controls [10–13]. Studies also showed that excessive levels of extracellular glutamate induce excitotoxicity and augment neuroinflammation and neuronal injury, which may play a role in the pathogenesis of HAND [14–19].

Glutaminase (GLS), a resident mitochondria enzyme, is specialized in the *de novo* synthesis of the neurotransmitter glutamate [20–24]. Two major types of GLS exist in mammals, which include “kidney-type” (GLS1) and “liver-type” (GLS2) transcribed from different genes. GLS has also been found to be abundant in the brain tissue [15]. In human brain, GLS1 has two allozymes: kidney-type glutaminase (KGA) [25] and glutaminase C (GAC) [26]. The allozymes are generated through tissue-specific alternative splicing from the same gene and have the identical core GLS1 enzyme domain but different 3′ tails [27]. Our previous studies suggested that GAC and KGA are differentially upregulated in HAND brain samples, HIV-1-infected MPs and inflammatory neurons [6, 28–31]. Increased extracellular levels of glutamate from activated MPs could cause excitotoxicity to neurons through NMDA receptor activation [28, 29]. Therefore, regulation of GLS1 isoforms is of importance to HAND research. A key molecular event associated with the elevation of glutamate is the release of GLS1 [28, 30, 31]. Although several early observations of GLS1 release were linked with cell death, more recent data from our lab suggested that mitochondrial stress could lead to membrane destabilization and relocation of GLS1 from the mitochondrial matrix to the cytosol through the permeability transition pore [32]. Because further release of GLS1 into extracellular supernatants contributes to excess glutamate production, it is imperative to understand the molecular mechanism of cellular GLS1 release.

Recent evidence indicates that microvesicles (MVs), unconventional cellular secretory vesicles, are shed from the plasma membrane and range from 100 nm to 1 μm in diameter [33]. Interestingly, MVs are abundant in the central nervous system (CNS) and are derived from multiple brain cell types, including neurons, microglia, oliogodendrocytes, and astrocytes [34]. Therefore, there is a growing appreciation of the important role of MVs in regulating the brain microenvironment [35, 36]. CNS-derived MVs may contribute to neuroinflammation through secretion of signaling molecules, nucleic acids, lipids, and proteins, and may participate in inter- and intra-cellular communication [33, 37–42]. Release of MVs is increased upon neural cancer progression, neuroinflammation, and acute neurological disorders. MVs could serve as a useful biomarker for CNS diseases including ischemic stroke, multiple sclerosis, glioblastoma, and other neurological and neurodegenerative disorders [43–45]. However, the role of MVs in the pathogenesis of neurodegenerative disorders, especially HAND, remains to be elucidated. In our current study, we identified MVs as a primary mechanism of GLS1 release, which subsequently mediates excess glutamate generation and neurotoxicity from HIV-1-infected macrophages and immune-activated microglia. The investigation of the function of GLS1-containing MVs is important for understanding a potentially pathological event in HAND, and it may provide possible therapeutic targets and a unique biomarker.

## Results

### HIV-1 infection and immune activation increase MV release from macrophages and microglia

**Fig. 1:**
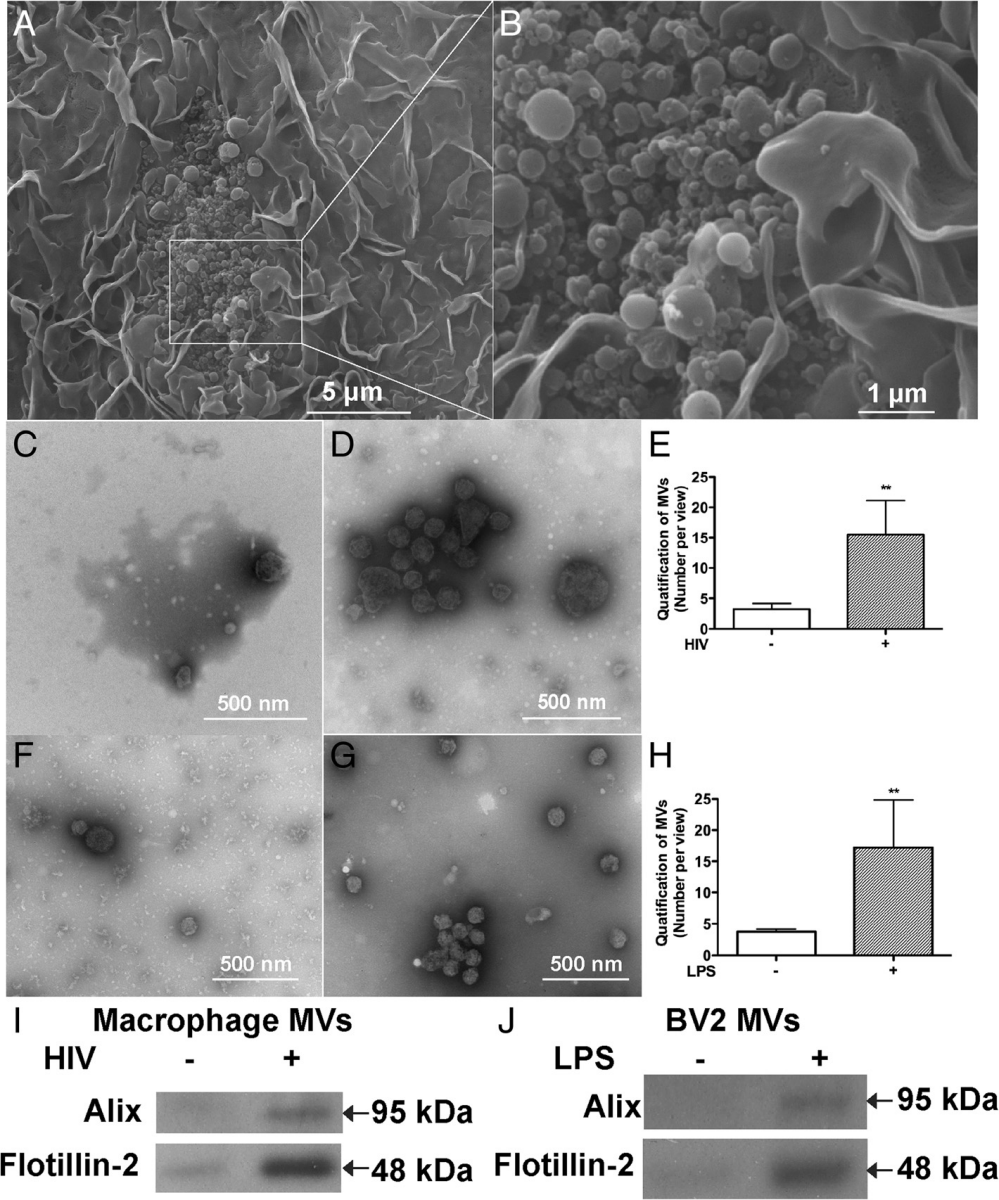
HIV-1 infection and immune activation increase MVs release from macrophages and microglia. **a** MDM were fixed at 7th day post HIV-1 infection and subsequently subjected to SEM for MV detection. Magnification, 6000 ×. **c** High-magnification image of the corresponding small box area in panel A was shown. Magnification, 24000 ×. **c**-**h** MVs were isolated through differential centrifugation from normalized volumes of cultural supernatants and observed under TEM using negative staining. Representative TEM images of MVs from mock-infected MDM (c), HIV-1 infected MDM (d), untreated microglia (f), and LPS-stimulated microglia (g) were shown. **e**, **h** MVs numbers in c, d and e, f were quantified by manually counting from a total of 10 random vision fields. Results are representative of TEM images and quantification results are means ± SD of MV numbers from 10 fields of TEM images. ** denotes *p* < 0.01 in comparison to controls. **i**, **j** MVs were isolated from normalized volumes of supernatants in mock-infected and HIV-1-infected MDM cultures (i) or untreated and LPS-stimulated microglia cultures (j). The levels of Alix and flotillin-2 in MVs were determined by Western blot

Our previous studies have demonstrated that GLS1 is released to the extracellular fluid during HIV-1 infection and neuroinflammation [28, 30, 31]. However, key molecular mechanisms that regulate GLS1 release remain unknown. Recent discovery of MVs during HIV-1 infection in macrophages and dendritic cells offers an exciting possibility that GLS1 may be released through MV mechanism [33, 46]. We used scanning electron microscopy (SEM) to identify MVs that were in the process of budding from macrophages (Fig. 1a, b). To quantitatively evaluate MVs release, two different techniques for MV detection were used. First, MVs were isolated from cultured monocyte-derived macrophages (MDM) supernatants by differential centrifugation. To reflect the changes of MVs from same number of cells, MVs were isolated from normalized volumes of supernatants based on the protein concentrations in the whole cell lysates. Cells, nuclei, debris and subcellular organelles were removed from supernatant after serial centrifugation. The MV pellet was collected and resuspended for negative staining under transmission electron microscopy (TEM). Images of MVs from ten random fields were captured. The numbers of MVs per field under TEM was significantly higher in HIV-1-infected macrophages than MVs from mock-infected macrophages, suggesting that HIV-1 infection leads to increased release of MVs in MDM (Fig. 1c-e). Similarly, when murine microglia cell line BV2 was treated with lipopolysaccharide (LPS) for immune activation, the MV number was significantly higher in the LPS-treated BV2 compared with the untreated group (Fig. 1f-h). Second, MVs isolated from infected human macrophages and immune activated microglia were subjected to Western blots for specific MV markers, including ALG-2 interacting protein (Alix) and flotillin-2. Consistent with TEM data, Western blot analysis revealed increased levels of Alix and flotillin-2 in MV lysates from HIV-1-infected cells, compared with those from uninfected cultures (Fig. 1i). The increased release of MVs from LPS-treated BV2 cells was further confirmed in Western blot by MV markers, Alix and flotillin-2 (Fig. 1j). Because MVs were isolated from normalized volumes of supernatants based on their corresponding whole cell protein concentrations, higher number of MVs in TEM and increased immunoreactivities of MV markers in Western blots from HIV-1-infected macrophages and LPS-stimulated microglia suggest that HIV-1 infection and immune activation both increase MV release from the cultures.

### MVs mediate GLS1 release in HIV-1-infected MDM and immune-activated microglia

**Fig. 2:**
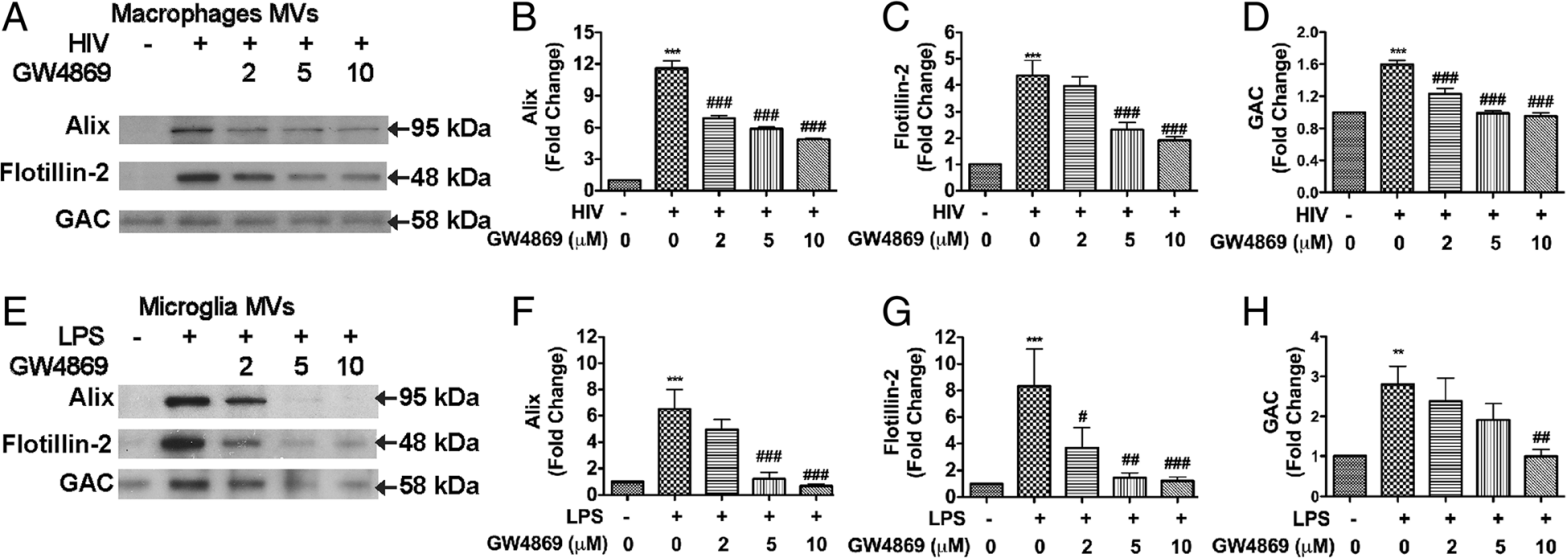
MVs mediate GLS1 release in HIV-1-infected MDM and immune-activated microglia. **a** At 7 days post-infection, mock-infected and HIV-1 infected MDM were treated with GW4869 for 24 h in serum-free media. MVs were isolated from the supernatants and MV protein lysates were prepared. The levels of Alix, flotillin-2, and GAC were determined by Western blots. **b**, **c**, **d** Densitometric quantifications of the Alix (b), flotillin-2 (c), and GAC (d) protein levels were presented as fold change relative to the mock-infected controls. **e** MVs were isolated from the supernatants of control and LPS-treated microglia in the presence and absence of GW4869. MV lysates were subjected to Alix, flotillin-2, and GAC detection through Western blots. Results shown are representative of three independent experiments. **f**, **g**, **h** Densitometric quantifications of the Alix (f), flotillin-2 (g), and GAC (h) protein levels were presented as fold change relative to the untreated controls. DMSO was used as solvent control for GW4869. Western blot results shown are representative of three independent experiments. Quantification results shown are means ± SD of experiments performed in triplicate (*n* = 3 donors). ** and *** denotes *p* < 0.01 and 0.001 in comparison to mock-infected or untreated control; #, ## and ### denote *p* < 0.05, 0.01 and 0.001 in comparison to HIV-infected or immune-activated groups, respectively

To determine whether MVs are the main mechanism for GLS1 release in HIV-1-infected MDM and immune-activated microglia, we treated the cultures with GW4869, a neutral sphingomyelinases (nSMase) inhibitor, for 24 h prior to MVs isolation. GW4869 is known to disrupt in the biogenesis of MVs [47, 48]. Because GW4869 was dissolved in DMSO, we used DMSO as a solvent control for GW4869 and used same amount of DMSO in each dose of the GW4869 treatment. MV markers, Alix and flotillin-2, were both increased in the MVs isolated from HIV-1-infected MDM (Fig. 2a-c). Pretreatment with GW4869 for 24 h reduced the levels of Alix and flotillin-2 in the MVs isolated from HIV-1-infected MDM, indicating the increased MV release in HIV-1-infected MDM could be blocked by GW4869 (Fig. 2a-c). The protein levels of GAC, a GLS1 isoform previously identified to have neurotoxic potential, were also increased in MVs after HIV-1 infection and diminished after GW4869 treatment, indicating that MVs carried GAC as cargo and that HIV-1 increases GAC release through MVs (Fig. 2a, d). Consistent with HIV-1 infection, LPS activation of microglia line BV2 cells also lead to increased MV secretion (Fig. 2e-g). After treatment with increasing concentrations of GW4869, the increase of Alix and Flotillin-2 in the MVs isolated from LPS-activated microglia was blocked, suggesting that immune activation also increases MV release in microglia (Fig. 2e-g). Importantly, GAC protein levels were also increased in MVs after LPS activation and diminished after GW4869 treatment, confirming that immune activation also increases GAC release through MVs (Fig. 2e, h).

### MVs isolated from HIV-1-infected MDM and immune-activated microglia mediate extracellular glutamate production through GLS1

**Fig. 3:**
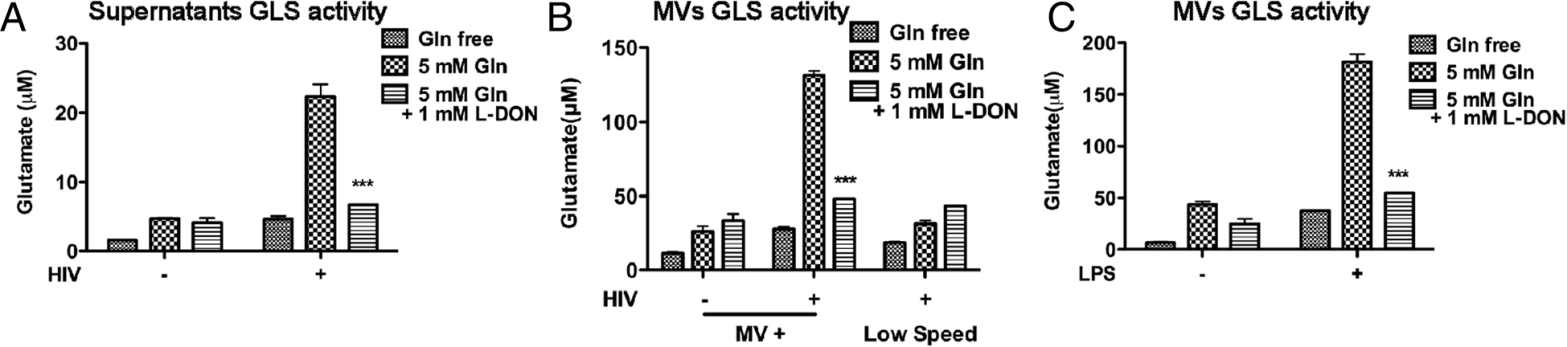
MVs isolated from HIV-1-infected MDM and immune-activated microglia mediate extracellular glutamate production through GLS1. **a** Cell-free supernatants from mock-infected and HIV-1-infected macrophages were incubated with or without glutamine and 1 mM L-DON *ex vivo* for two days. The resulting glutamate levels were determined by RP-HPLC. *** denotes *p* < 0.001 in comparison to the 5 mM glutamine group in HIV-1-infected samples. **b** MVs were isolated from cell-free supernatants from mock-infected and HIV-1-infected MDM and incubated with or without glutamine and 1 mM L-DON *ex vivo* for two days. Glutamate generation in MVs or low speed (LS) pellets was determined by RP-HPLC. Low speed pellets were collected after 10,000 g centrifugation, which contained subcellular organelles and debris. *** denotes *p* < 0.001 in comparison to 5 mM glutamine group in HIV-1-infected samples. **c** MVs were isolated from cell-free supernatants from untreated and LPS-activated microglia and incubated with or without glutamine and 1 mM L-DON *ex vivo* for two days. Glutamate generation in MVs was determined by RP-HPLC. *** denotes *p* < 0.001 in comparison to 5 mM glutamine group in LPS-activated samples. Results are means ± SD of triplicate samples and are representative of three independent experiments

The identification of MVs in mediating GAC release raises the question of functional relevance of GAC to MVs. HIV-1 infection is known to increase extracellular levels of glutamate in human macrophages [2]. We treated cell-free HIV-1-infected supernatants with 5 mM glutamine, the reaction substrate for GLS enzyme, in the presence of 6-Diazo-5-oxo-L-norleucine (L-DON), a GLS1 inhibitor. The levels of glutamate production, as determined by RP-HPLC, increased after HIV-1 infection, which was dependent on glutamine (Fig. 3a). Furthermore, the elevation of glutamate was blocked by L-DON, indicating that the released GLS1 promotes glutamate generation in the extracellular fluid of HIV-1-infected MDM (Fig. 3a). More importantly, MV pellets extracted from both mock- and HIV-1-infected MDM were directly tested for GLS activity by HPLC. MVs were incubated with neurobasal media with or without glutamine for two days. Interestingly, the levels of glutamate generated in MVs from HIV-1-infected cultures elevated significantly compared with mock-infected control (Fig. 3b). Low speed pellets were collected after 10,000 g centrifugation, which may contain subcellular organelles and debris. Interestingly, blockage of GLS1 activity by L-DON was only observed in the MVs but not in the low speed pellets from HIV-1-infected MDM, suggesting that MVs are the specific compartments for GLS1 release and responsible for excess glutamate production (Fig. 3b). Consistent with HIV-1-infected MDM, MVs isolated from supernatant of LPS-stimulated microglia significantly increased glutamate generation, which could be blocked by L-DON (Fig. 3c). To further investigate how MVs facilitate glutamine hydrolysis through the GLS1, we determined the level of vesicular glutamate transporter (VGLUT) in the MV lysates from HIV-1 infected MDM through Western blots (Additional file 1: Figure S1). VGLUT levels increased after HIV-1 infection and decreased after GW4869 treatment, consistent with the overall MV levels. These results indicate VGLUT is a component of MVs and suggest that glutamate transporters on the MV lipid bilayer may facilitate the transportation of glutamate. Taken together, these data suggest that GLS1 released through MVs in HIV-1-infected MDM and immune-activated microglia maintains enzyme activity and promotes glutamate generation.

### GLS1-containing MVs induce neurotoxicity

**Fig. 4:**
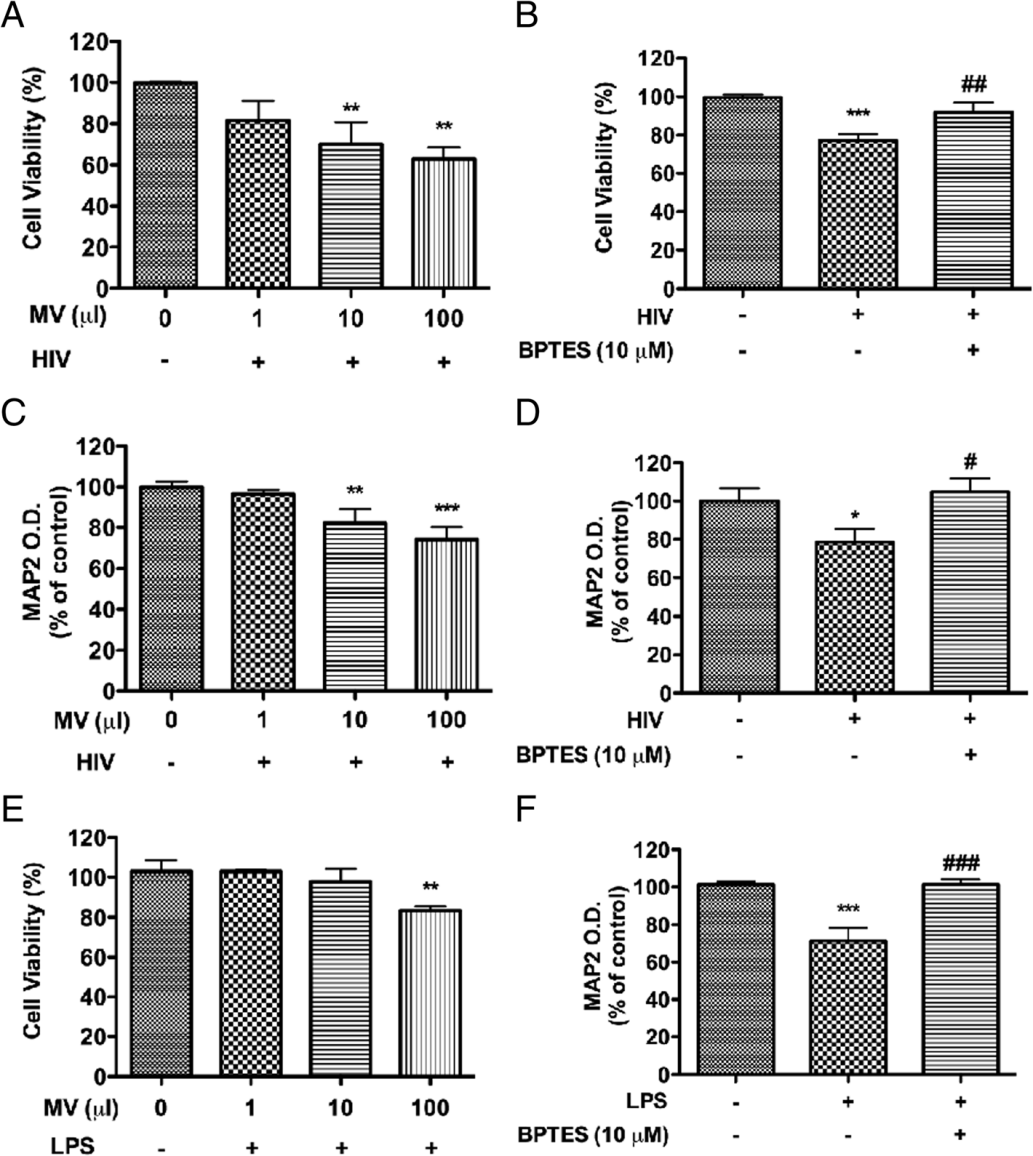
GLS1-containing MVs induce neurotoxicity. **a**-**d** Extracellular MVs were isolated from mock-infected and HIV-1-infected MDM at 7 days post-infection and incubated with RCN at the indicated dosage for 24 h (a, c). 100 μl of MVs were added in RCN with or without 10 μM of BPTES, a GLS1 inhibitor, for 24 h (b, d). Neurotoxic potentials of MVs were determined by MTT (a, b) and MAP2 ELISA assays (c, d). **e**, **f** Extracellular MVs were isolated from control and LPS-activated microglia and incubated with RCN with or without 10 μM of BPTES for 24 h. Neurotoxic potentials of MVs were determined by MTT with incubation of different dosages of MVs (e) and MAP2 ELISA assays with incubation of 100 μl of MVs (f). *, ** and *** denote *p* < 0.05, 0.01 and 0.001 in comparison to controls, respectively; #, ## and ### denote *p* < 0.05, 0.01 and 0.001 in comparison to HIV-infected group, respectively. Results are expressed as means ± SD of triplicate samples and are representative of three independent experiments

To investigate the functional significance of GLS1-containing MVs from HIV-1-infected MDM and immune-activated microglia, MVs were collected and re-suspended in neuronal culture medium. The volumes of culture medium used to re-suspend MVs were adjusted based on the whole cell protein concentration in the same culture. Rat cortical neurons (RCN) treated with 10 μl or 100 μl per well of MVs from HIV-1-infected MDM had significantly lower viabilities (Fig. 4a) and higher neurotoxicity (Fig. 4c) compared with those treated with MVs from mock-infected cells. However, the MV-induced toxicity was rescued at the presence of BPTES, a GLS1 inhibitor, when 100 μl of MVs were incubated with RCN (Fig. 4b, d). Similar neurotoxicity was observed in neurons treated with MVs isolated from LPS-activated microglia (Fig. 4e), an effect that was blocked by BPTES (Fig. 4f). These data suggest that the GLS1-containing MVs induce neurotoxicity through the GLS1 activity in the MVs.

### HIV-1-infected MDM induce neurotoxicity through GLS1-containing MVs

**Fig. 5:**
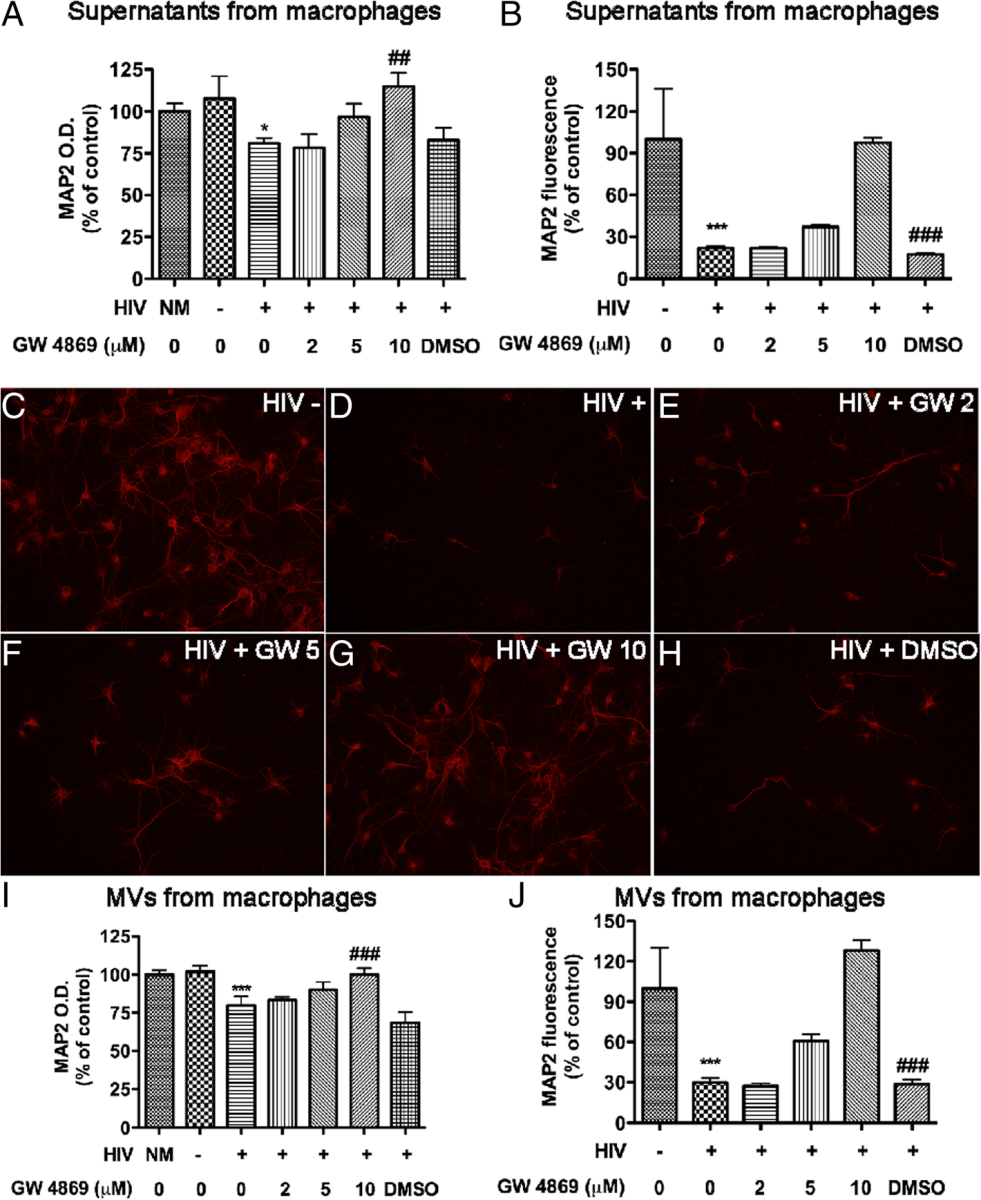
HIV-1-infected macrophages induce neurotoxicity through GLS1-containing MVs. At 7 days post-infection, mock-infected and HIV-1 infected macrophages were treated with GW4869 at different dosages for 24 h. Cell-free supernatants and MVs were collected and added to RCN cultures for neurotoxicity. DMSO was used as solvent control for GW4869. **a**, **b** Neurotoxic potentials of the supernatants were determined by MAP2 ELISA assay (a) and quantification of MAP2 fluorescence in immunostaining (b). **c**-**j** Neurotoxic potentials of MVs were determined by MAP2 ELISA assay (i) and immunostaining (c-h) followed by quantification of the intensity of MAP2 fluorescence after MVs treatment. NM in panels A and I stands for neuronal media. * and *** denote *p* < 0.05 and 0.001 in comparison to mock-treated control, respectively; ## and ### denote *p* < 0.01 and 0.001 in comparison to DMSO-pretreated HIV-infected group, respectively. Results are expressed as means ± SD of triplicate samples and are representative of three independent experiments

To determine whether HIV-1-infected MDM induce neurotoxicity through GLS1-containing MVs, we obtained supernatants from HIV-1-infected macrophages that were pre-treated with 2, 5 or 10 μM of GW4869 and used the supernatants to treat RCN for neurotoxicity. Similar with prior experiments, we used DMSO as a solvent control for GW4869 and used same amount of DMSO in each dose of the GW4869 treatment. Neurotoxicity was determined through quantifications of neuronal antigen MAP2 with either MAP2 ELISA (Fig. 5a) or MAP2 immunostaining (Fig. 5b, Additional file 2: Figure S2). The HIV-1-infected MDM-induced neurotoxicity was blocked by increasing concentrations of GW4869 (Fig. 5ab), indicating that supernatants from HIV-1-infected MDM induce neurotoxicity through MVs. Next, we isolated MVs from mock-infected and HIV-1-infected MDM and investigated the direct neurotoxicity of MVs. Interestingly, MVs isolated from HIV-1-infected MDM manifested higher levels of neurotoxicity as determined by MAP2 ELISA (Fig. 5i) or MAP2 immunostaining (Fig. 5c-h, j), when compared with MVs from mock-infected control. Treatment of GW4869 prior to MV isolation rescued MV-induced neurotoxicity at a dose dependent manner, suggesting a direct neurotoxic role of MVs from HIV-1-infected MDM. Together, these data strongly suggest that HIV-1-infected macrophages induce neurotoxicity through MVs.

## Discussion

Our previous reports have described the release of GLS1 into extracellular space during neuroinflammation or HIV-1 infection. However, key molecular mechanisms that regulate GLS1 release remain unknown. Our current study presents two important new findings regarding GLS1 release. First, MVs contain GLS1, which is a key enzyme for generating glutamate in the brain, and GLS1 is released into the extracellular fluid primarily via MVs in HIV-1-infected cells and immune-activated microglia. Second, HIV-1 infection and LPS activation increase the magnitude of MV release from macrophages and microglia. Interestingly, increased release of GLS1-containing MVs also induces excitotoxicity in RCN. The toxic effect of MVs was reversed by glutaminase inhibitors and MV inhibitors. These observations suggest that MVs may contribute to excess glutamate production in macrophages in the context of HIV-1 neurotoxicity.

The physiological relevance of this observation is significant, where elevated endogenous levels of GLS1 have been reported in the post mortem brain tissues of HIV-1-associated dementia patients [29, 49]. Furthermore, it has been demonstrated that both of the upregulated GLS1 isoforms, KGA and GAC, are released from the inner membrane of mitochondria into the cytosol through the permeability transition pore [31, 32]. However, it is the extracellular glutamate that causes neurotoxicity, and the mechanisms by which cytosolic GLS1 is released into the cell supernatant have not been previously established. The current study provides strong evidence that GLS1-containing MVs are the main mechanism for the release of GLS1 from the macrophage and microglia cytosol into the extracellular compartment, where the extracellular glutamate subsequently induces neurotoxicity. It is unclear how GLS1 hydrolyzes extracellular glutamine inside MVs. We have detected vesicular glutamate transporter in the MV lysates from HIV-1 infected MDM (Additional file 1: Figure S1), suggesting that MVs may transport glutamate across its lipid bilayer through glutamate transporters.

Our studies were designed to rigorously establish the purity and characterization of the isolated microvesicles. To confirm complete separation of MVs from mitochondria, the absence of the mitochondrial marker protein voltage-dependent anion-selective channel and cytochrome C was confirmed through Western blots (data not shown). Secondly, to characterize the MVs, both scanning and transmission electron microscopies were used, which showed vesicles ranging in size from 100 nm to 500 nm. Western blot could not detect the presence of GLS1 in exosomes collected from commercially available exosome kits, suggesting that GLS1 is selectively packed into MVs rather than into smaller exosomes. Furthermore, because the MV-free supernatants and the pellets that contained cellular debris had minimum GLS activities and GLS1 was found predominantly in the isolated MVs fraction, we concluded that MVs are the primary instigator in facilitating GLS1 release in HIV-1-infected macrophages and LPS-activated microglia. Chronic activation of the immune system is a hallmark of progressive HIV infection yet its etiology remains obscure. Circulating microbial products such as LPS, possibly derived from the gastrointestinal tract, was significantly increased in chronically HIV-infected individuals and may be a cause of HIV-related systemic immune activation [50]. In our report, when LPS was used to treat BV2 microglia cell line, high levels of GLS1 were found in the supernatants leading to neurotoxicity. These results support the pathogenic effect of the immune activation in the CNS during HIV-1 infection. The discovery that immune activation induces GLS1 release via MVs may have a broader implication to other neurological diseases, where excitotoxicity and GLS1 are involved. Because MVs are abundantly expressed in the CNS, it is tempting to speculate whether qualitative or quantitative changes of MVs contribute more broadly to neurological diseases. In addition, the regulation of MVs could be exploited as a novel therapeutic target.

In the current study, we also utilized a MV inhibitor, GW4869, to block the release of the MVs. Both GLS1 and MV markers were decreased in a dose-dependent manner by GW4869, suggesting that GLS1 release is through MVs. GW4869 is an inhibitor for nSMase2, which is responsible for the production of ceramide. Ceramide has been found enriched in MVs and involved in the formation of vesicles. Our results point to a possible mechanism of MVs release in the microglia and macrophages through the endosomal sorting complex required for transport (ESCRT) machinery or a ceramide-dependent pathway [47, 48, 51]. These interesting possibilities remain the subject of future investigation. Furthermore, we have demonstrated that the neurotoxicity by HIV-1-infected MDM was abolished by pre-treatment with GW4869, which indicates that GLS1-containing MVs are the neurotoxic factors in HIV-1-infected macrophages and immune-activated microglia. Therefore, inhibiting the release of MVs might become a potential therapeutic approach for the treatment of HAND patients.

Three aspects of the studies merit further investigation. First, how is cytosolic GLS1 loaded into MVs for extracellular secretion from macrophages? Second, how are GLS1-containing MVs formed? Third, what mediates the release of GLS1-containing MVs from the plasma membrane? Autophagosomes, inflammasomes or mitochondria-derived vesicles may provide possible mechanisms for these events to occur. It is possible that HIV-1 infection and immune activation of macrophages and microglia directly lead to release of GLS1-containing MVs, however, other possibilities also need to be explored.

## Conclusions

In summary, our studies address important questions regarding the cellular mechanisms of GLS1 release, implicating a critical role of MVs. This newfound knowledge of neurotoxic GLS1-containing MVs has potentially important clinical implications for neurologic diseases such as HAND. Developing inhibitors of MV formation and release or inhibitors of GLS1 might yield effective new therapies for reducing glutamate-induced neurotoxicity in HIV-1-infected and immune-activated individuals.

## Methods

### Culture, HIV-1 infection and LPS activation of macrophages and microglia

Human peripheral blood-derived mononuclear cells were isolated through leukopheresis from healthy donors. Human macrophages were differentiated in Dulbecco’s Modified Eagle’s Media (DMEM) (Sigma Chemical Co., St. Louis, MO) with 10 % human serum, 50 μg/ml gentamycin, 10 μg/ml ciprofloxacin (Sigma), and 1000 U/ml recombinant human macrophage colony-stimulating factor (MCSF) for 7 days. The HIV-1_ADA_ strain was used to infect the macrophages at a multiplicity of infection (MOI) of 0.1, respectively. The HIV-1_ADA_ strain was originally isolated from the PBMCs of an HIV-infected patient with Kaposi’s sarcoma [52, 53]. MDM were infected with HIV-1_ADA_ at a multiplicity of infection (MOI) of 0.05 virus/target cell. For mock-infection, MDM were incubated with same volume of medium without virus. After 24 h, the culture medium was changed to remove any remnant virus. Seven days after HIV-1-infection, culture medium was changed to glutamine-free neurobasal medium for 24 h and supernatants were collected for subsequent RP-HPLC or Western blot analysis. BV2 cell lines were obtained from ATCC, and both cell lines were grown in DMEM with 10 % fetal bovine serum and antibiotics. Lipopolysaccharide (LPS) (50 ng/ml) (Sigma) was used to immune activate BV2 cells for 24 h and supernatants were collected for HPLC and Western blot analysis.

### Rat cortical neuron cultures

Cerebral cortices were dissected from Sprague–Dawley rat (Charles River Laboratories International Inc., Wilmington, MA) between embryonic days 15 and 17 and triturated with a pipet to generate cell suspension. The cell suspension was passed through a 70-μm nylon membrane (Becton Dickinson Labware, Franklin Lakes, NJ) and then plated at a density of 40,000 cells/well in 96-well plates pre-coated with 5 μg/ml poly-D-lysine. The cells were then cultured at 37 °C in a 5 % CO_2_ atmosphere for 7 days in neurobasal medium containing B27 supplement (Life Technologies), 0.5 mM glutamine, 100 U/ml penicillin and 100 μg/ml streptomycin.

### Ethics statement

Primary RCN were prepared in accordance with ethical guidelines for care and use of laboratory animals set forth by the National Institutes of Health (NIH), with Institutional Animal Care and Use Committee (IACUC) #: 04-097-01; Monocytes were used in full compliance with the University of Nebraska Medical Center and NIH ethical guidelines, with the Institutional Review Board (IRB) #: 162-93-FB. We have the informed written consent from all participants involved in this study.

### Isolation of MVs

MVs were isolated from the supernatants of HIV-1-infected macrophages and LPS-activated microglia through differential centrifugations with or without neutral sphingomyelinases inhibitor GW4869 (Sigma) at different dosages, 2, 5 and 10 μM. Briefly, the supernatants were first centrifuged at 300 × g for 10 min to remove free cells, at 3000 × g for 20 min to remove cellular debris and then 10,000 × g for 30 min to remove free organelles. Lastly, MVs were collected by ultracentrifugation at 100,000 × g for 2 h at 4 °C. To prepare MVs for Western blot, the MVs pellets were lysed in M-PER mammalian protein extraction reagent (Thermo Scientific, Pittsburgh, PA). For negative staining, MVs were fixed in 2 % glutaraldehyde and 2 % paraformaldehyde. For glutaminase activity assay and neurotoxicity, the MVs were resuspended in 1 ml of glutamine-free neurobasal medium.

### Negative staining and electron microscopy

MVs were negatively stained with onscreen measurements. Briefly, MVs were fixed and then spread on the silicon monoxide and nitro-cellular film coated copper grid. The droplets of MVs were removed with filter paper, air-dried at room temperature and then subjected to transmission electron microscopy (TEM) (FEI Tecnai G2 Spirit TWIN). For the scanning electron microscope (SEM) (FEI Quanta 200), cells were fixed in 2 % glutaraldehyde and 2 % paraformaldehyde and point dried, mounted and coated with gold/palladium. The investigator in the EM core facility was blinded for image acquisition and quantification.

### Neurotoxicity assays

Resuspended MVs were added to neuronal cultures for 48 h with or without 10 μM of bis-2-(5-phenylacetamido-1,2,4-thiadiazol-2-yl)ethyl sulfide (BPTES) (a generous gift presented by Dr. Tsukamoto from Colorado State University) and cell viability was assessed by MTT assays in 96-well plates. MTT (Sigma) was added to the cultures to a final concentration of 125 μg/ml. The plates were incubated for 30 min at 37 °C with 5 % CO_2_ and the medium was aspirated. The insoluble formazan was solubilized in DMSO, and the concentrations were determined by optical density at 490 nm with an EL_X_808 densitometer (Bio-Tek Instruments, Winooski, and VT). MAP2 ELISA was performed on primary RCN cultures as previously described. Briefly, fixed neurons were blocked with 3 % normal goat serum in phosphate buffered saline and incubated for 2 h with antibodies against MAP-2 (Millipore-Chemicon International, Atlanta, GA), followed by anti-mouse biotinylated antibody (Vector Laboratories, Burlingame, CA) for 1 h. Avidin/biotin complex solution was added for 30 min, and then color was developed using TMB substrate (Sigma Chemical Co., St. Louis, MO) and terminated with 1 M sulfuric acid (Sigma Chemical Co., St, Louis, MO). The absorbance was read at 450 nm using a microplate reader (Bio-Rad Laboratories, Hercules, CA). For morphological data that demonstrated neuronal damage after exposed to supernatant of HIV-1-infected macrophages or immune-activated microglia, MAP2 immunostaining was examined by a Nikon Eclipse TE2000E fluorescent microscope and photographed by a digital camera (CoolSNAP EZ, Photometrics). All obtained images were imported into Image-ProPlus, version 7.0 (Media Cybernetics, Sliver Spring, MD) for quantifying levels of MAP2 staining. The assessors were blinded during image acquisition or quantification.

### Western blot

Protein concentrations were determined by Bradford protein assay. SDS PAGE separated proteins from whole cell and MVs lysates. After electrophoretically transferred to polyvinyldifluoridene membranes (Millipore, Billerica, MA and Bio-Rad, Hercules, CA). Membranes were incubated overnight at 4 °C with polyclonal antibodies for GAC (Dr. N. Curthoys, Colorado State University, Fort Collins, CO), Alix (Santa Cruz Biotechnology, CA) and flotillin-2 (Cell Signaling Technology, Danvers, MA), followed by horseradish peroxidase-linked secondary anti-rabbit or anti-mouse secondary antibodies (Cell signaling Technology). Antigen-antibody complexes were visualized by Pierce ECL Western Blotting Substrate. For quantification of the data, films were scanned with a CanonScan 9950 F scanner and images were analyzed using the public domain NIH image program (developed at the U.S. National Institutes of Health and available on the internet at http://rsb.info.nih.gov/nih-image/).

### Analysis of glutamate and glutamine by RP-HPLC

Glutamate levels were analyzed by RP-HPLC using an Agilent 1200 liquid chromatograph and fluorescence detector as previously described [29] with a few modifications. The experiments utilized 4.6 × 75 mm, 3.5 μm ZORBAX Eclipse AAA analytical columns (Agilent). A gradient elution program was optimized for glutamate measurement with a flow rate 0.75 ml/min.

### Statistical analysis

Data are expressed as means ± SD unless otherwise specified. Statistical analysis was performed using ANOVA, followed by the Tukey-post-test for paired observations. Significance was determined by a *p* value < 0.05. All experiments were performed with cells from at least three donors to account for any donor-specific differences. Assays were performed at least three times in triplicate or quadruplicate within each assay.

## Additional files

Additional file 1: Figure S1. MVs from HIV-1-infected MDM contain vesicular glutamate transporter. At 7 days post-infection, mock-infected and HIV-1 infected MDM were treated with GW4869 for 24 h in serum-free media. MVs were isolated from the supernatants and MV protein lysates were prepared. The levels of vesicular glutamate transporter were determined by Western blot. (TIFF 41 kb) Additional file 2: Figure S2. HIV-1-infected macrophages induce neurotoxicity through GLS1-containing MVs. (A-F) At 7 days post-infection, mock-infected and HIV-1 infected macrophages were treated with GW4869 at different dosages for 24 h. Cell-free supernatants were collected and added to RCN cultures for neurotoxicity. DMSO was used as solvent control for GW4869. Neurotoxic potentials of the supernatants were determined by MAP2 fluorescent staining. Results are representative of 20 fluorescent images from three independent experiments. (TIFF 523 kb)

## Abbreviations

BPTES: bis-2-(5-phenylacetamido-1,2,4-thiadiazol-2-yl)ethyl sulfide
CNS: central nervous system
GAC: glutaminase C
GLS: glutaminase
GLS1: glutaminase 1
HAND: HIV-1-associated neurocognitive disorders
KGA: kidney-type glutaminase
L-DON: 6-Diazo-5-oxo-L-norleucine
LPS: Lipopolysaccharide
MPs: mononuclear phagocytes
MVs: microvesicles
RP-HPLC: reverse phase high-performance liquid chromatography
SEM: scanning electron microscopy
TEM: transmission electron microscopy
