# Supplementary figures and images for: Glutaminase-containing microvesicles from HIV-1-infected macrophages and immune-activated microglia induce neurotoxicity
Source: Mol Neurodegener. 2015 Nov 6;10:61. doi: 10.1186/s13024-015-0058-z (PMC4635976; doi:10.1186/s13024-015-0058-z)

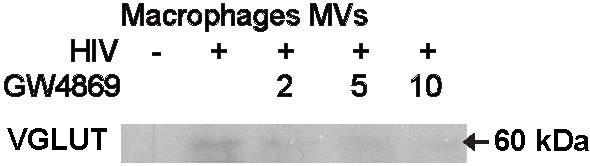

Supplement: Additional file 1: Figure S1. — MVs from HIV-1-infected MDM contain vesicular glutamate transporter. At 7 days post-infection, mock-infected and HIV-1 infected MDM were treated with GW4869 for 24 h in serum-free media. MVs were isolated from the supernatants and MV protein lysates were prepared. The levels of vesicular glutamate transporter were determined by Western blot. (TIFF 41 kb) [file 13024_2015_58_MOESM1_ESM.tiff]

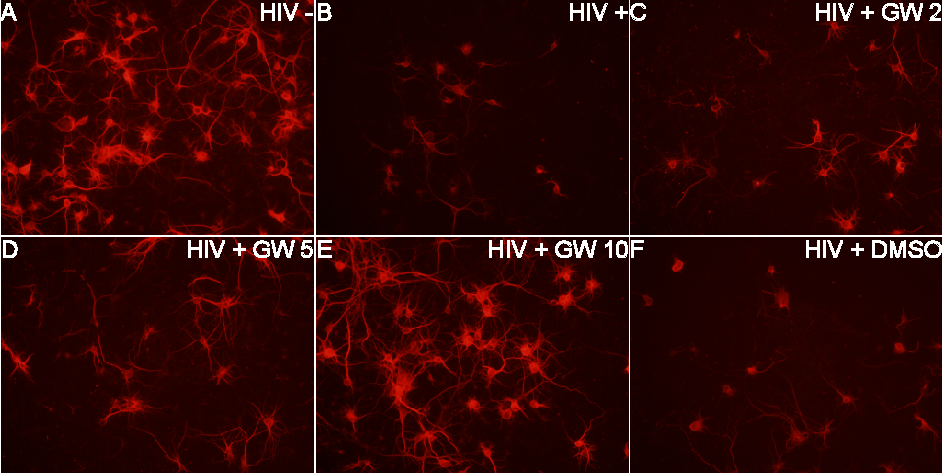

Supplement: Additional file 2: Figure S2. — HIV-1-infected macrophages induce neurotoxicity through GLS1-containing MVs. (A-F) At 7 days post-infection, mock-infected and HIV-1 infected macrophages were treated with GW4869 at different dosages for 24 h. Cell-free supernatants were collected and added to RCN cultures for neurotoxicity. DMSO was used as solvent control for GW4869. Neurotoxic potentials of the supernatants were determined by MAP2 fluorescent staining. Results are representative of 20 fluorescent images from three independent experiments. (TIFF 523 kb) [file 13024_2015_58_MOESM2_ESM.tiff]
